# Capturing The Pain Experience: Creation and Testing of an AI-Driven Pain Assessment App

**DOI:** 10.1093/geroni/igaf122.3724

**Published:** 2025-12-31

**Authors:** Marcia Shade, Mrinal Rawool, Stephen Scott

**Affiliations:** University of Nebraska Medical Center, Omaha, Nebraska, United States; University of Nebraska-Lincoln, University of Nebraska Lincoln, Nebraska, United States; University of Nebraska-Lincoln, University of Nebraska Lincoln, Nebraska, United States

## Abstract

**Background:**

Pain is undertreated and poorly managed in older adults. Pain symptoms may not be reported or identified, and time is limited in clinical practice to address pain among multiple comorbid conditions. Can artificial intelligence assist? The purpose of this project was to create and test a machine learning model (MLM) that captures the remote biopsychosocial pain experience.

**Methods:**

Mock data interactions were used to fine-tune a pre-trained model. The model was integrated with a mobile software application’s architecture and deployed for testing. Metric data was collected on the MLM’s response time, crash, and transcription grammatical accuracy.

**Results:**

The MLM generated pain assessment questions, such as, Is the pain worse at certain times of the day, like mornings or evenings? During the initial model creation, synthetic conversation testing yielded ROUGE scores of 0.5–0.6 and BLEU scores of 0.62. During testing, the MLM’s average response time was 4 seconds per request. Requests were impacted slightly by the length of the spoken message from the user. One app crash occurrence was documented. The MLM was grammatically accurate; however, the user utterance transcription grammatical error rate was 1.2%.

**Conclusions:**

These findings demonstrate the promising metrics from iterative development, deployment, and testing of an MLM designed to engage with older adults with chronic pain. The response time underscores the preliminary efficiency of this integrated system. Continued refinement of the model with performance and user experience testing in real-world settings will help optimize this solution.

